# Diseased Kindneys in Scarlatina Experimental Studies Thereon

**Published:** 1871-09

**Authors:** Carl Proegler

**Affiliations:** Aurora, Ill.


### DISEASED KIDNEYS IN SCARLATINA—EXPERIMENTAL STUDIES THEREON.

BY DR. CARL PROEGLER, Aurora, Ill.

During two epidemics of Scarlatina in New York State, I found that as late as up to the sixth or seventh week of continued illness, a great "desquamation" of the whole body of the patient took place. They were extreme hard cases, and combined with disease of the kidney. As soon as desquamation began, "albuminuria" and "hydrops" disappeared, and the patient got well. I concluded therefore to see whether there was a closer connection between the disease of the skin and kidney in Scarlatina, as there is at the present time thought of.

I used for the experiments rabbits. I suppressed the function of the skin by painting them partly over either with oil, gum, varnish, &c., or to produce inflammation of the skin I used croton oil and turpentine. I experimented on 13 rabbits. Two of them died after 40 hours, four in the first 24 hours. With the first six I noticed four times "albuminuria," once the urine was free from "albuminuria," and once I could not get any urine. The animals died, all with the same symptoms. Respiration, first superficial and quick, began to get slower and deeper. The extremities and ear cold, and death ensued by convulsions. The anatomical changes were the following:—the skin showed on the places where it was painted over, very large and strong injected vessels; greater and smaller petechias; subcutaneous tissues very moist; the inner organs very sanguinous; the right heart contained dark and badly coagulated blood. The kidneys, which were very dark and sanguinous, by examination did not show any change of tissue by microscopic examination. Four others of the rabbits died in the course of six and nine days; one on the third. In four cases albuminuria was very great, the filtered urine showed but few morphotic substances, some epithelium and lymph globules, only in one instance they were more abundant, and appeared to be more transparent in cylindrical shaped form of pale opaque color. One of the animals died with pleuro-pneumonia on the right side, and strange enough, without any albuminuria.

In the four cases the following were the changes of the kidney, they seemed somewhat larger than normal. The outer surface smooth, the capsule easily to divide. The substance very dark and sanguinous, the "corticulis" dark redish-brown, interlined on some places with pale yellow stripes. The papilla of light rose color. The microscopic examination revealed the epithelium of the tubuli uriniferi somewhat shaded and enlarged. The glomeruli were very sanguinous, therefore not very transparent. The epithelium of the capsular dilatation of the malphigian body were normal. The epithelium of the straight tubes were normal, transparent, and well preserved. The bloodvessels of the "medullar substance" very strongly filled with blood. In the interstial tissue of the kidney nothing abnormal could be found in the three cases; in the fourth case the interstitial tissues of the kidney were filled with dense masses of partly round and partly long-shaped corpuscles, the first a little smaller than white blood corpuscles, but with the same appearance. The central part of them could not be discerned.

Of the two last rabbits, one lived 15 the other 19 days. They both had on the first day "albuminuria." The residue of the urine showed "lymphoide corpuscles," with thick and red cells (dead epithelium) and red blood corpuscles; fatty and cylinder-shaped conglomeration, could not be found. The kidneys of the first animal (15 days) was very sanguinous, and the microscopic examination revealed the same facts as already mentioned, with the only exceptions that the "glomeruli" looked pale and bloodless, and that the free capillaries of the outer surface of the kidney were strongly filled with blood. The animal dying last, the kidneys were pale, the cortical substance wider than usual, and of a dirty yellow-gray color. In the last instance the epithelium of the "tubuli uriniferi" was not very clear and not transparent. The tubes seemed to be filled up with a dark mass resembling fine coarse sand. Bodies of destroyed epithelium could be seen. No fatty degeneration. In the straight tubes the epithelial cells were well preserved and transparent. No interstitial changes of any amount.

By my experiments I draw the following conclusions:—"Large inflammatory diseases of the skin will lead to diseased changes of the kidneys, first to active hyperæmia, which will, by long duration, give rise to parenchymatous and interstitial anomalies of the tissues." Taking these results with the experiments in scarlatina together, we will find that at least part of diseased kidneys in scarlatina are produced not only primarily by the "scarlatinous poison," but secondary in consequence of "scarlatinous dermatitis." In healthy adults skin and kidneys will sustain each other. But in scarlatina the inflamed skin cannot fulfil its function, but how is not yet quite clear, and the whole function is thrown upon the kidneys. This causes "hyperæmia," and, in longer duration, inflammatory changes of the kidney. It has been very often noticed that where "albuminuria" could be detected the secretion of urine is greater. But the beginning and cause of scarlatinous diseased kidneys has not revealed anything specific. Thomas (Virchow's Archiv.) tried to explain some "cylindroide," but they can be found with any catarrhal symptoms of the urinary ducts. That they originate from the kidneys, on account of their curious shape is not very probable, and their relations with the ureters, on account of the "microchemical properties," rather dubious. If you add highly diluted hydrochloric acid to the urine, it will not dissolve, but add to it a little concentrated nitric acid they disappear, more resembling therefore the "mucus" than the "albumin." I never noticed changes of the cylindroide on the "ureters." I take them therefore for curious-shaped masses of mucus, and have for the kidney in scarlatina no practical bearing. But why scarlatina is so often complicated with kidney disease, more so than in "acute dermatitis," is at the present moment not easy to say. The reason may be, that scarlatina covers the skin fully, unlike "morbillen" and "variola," where one may find between their efflorescence yet sound skin. My experiment shows that the skin in scarlatina claims our first attention. Baths and ointments of "glycerine salve" or Cologne water to be used externally, with the use of supoforics internally, etc. Belladonna, which has been claimed almost as a specific, has entirely failed in my hand.

